# 

**Published:** 1854-10-01

**Authors:** 


### To Correspondents.

Owing to the unexpected length of two of the articles preceding Dr. Webster's communication, we have been reluctantly compelled to defer the publication of several reviews, &c., now in type, until the next number of our Journal. We hope in that number to notice all the books, pamphlets, and periodicals, transmitted to us. Our analysis of the "Asylum Journal" is among the postponed articles. Dr. Jarvis's communication, forwarded to the Editor by the instruction of the Commissioners of Lunacy for the State of Massachusets, U. S., America, has been received and will be attended to. The last three numbers of the "American Journal of Insanity" will be reviewed in our January number, with the French Annals of Medical Psychology.

The concluding part of the Editor's Lettsomian Lectures, "On Medico-legal Evidence in Cases of Insanity," will appear in our January number.

### INDEX TO VOL. VII.

ACUTE mania, its characteristics, 28
Advocates, duties of, 398
American asylums for the insane, 273
Ancients, their knowledge of astronomy, 5
Apoplexy, treatment of, 331
Art, present state of in England, 509
Artistic Anatomy, by Robert Knox, M.D., 504
Astrology, its uses, 5—7
Asylums, position of, 94
Austria, institutions for the insane in, 443
Autobiography of the Rev. William Walford, 356

Baillarger, M., on the classification of mental diseases, 531
Bathing, efficacy of, in cases of acute mania, considered, 229
Bennett, Duke of Manchester *v.*, case of, 598
Blood sympathies, considered, 166
Body, influence of the mind on the, 83
Brain, its connexion with conscious life, 511
——— its weight and specific gravity in insanity, 201
Britons, their taste in art, 507
Brough, the Queen *v.*, case of, 609

Cahagnet, L. A., on magnetism, 301
Capital punishment, 43
Cellulose, chemical reaction of, in the brain of Man, by Rudolph Virchow, 284
Cerebellum, functions of the, 102
Cerebral ganglia, laws and action of, considered, 161
Cerebrum, its anatomical and psychological position, considered, 160
Charlesworth, Dr., on the non-restraint system, 151
Children, responsibility of parents for their crime, 39
Chorea, pathology of, considered, 338
Close, Rev. F., on table-turning, 18
Colon, disease of, its connexion with insanity, 172
Coma, renal epileptic, treatment of, 332
Commissioners in lunacy, their remarks on the management of asylums, publication of, 96
Consciousness, suspended, 497
Con-sensual ganglia, 513
Crime, its causes and remedies, by Frederick Hill, 35
——— removal of incitements to, 38
——— the hygiene of, 35, 50
Criminal cases, functions of the jury in, 199
————— plea of insanity in, 184
——— lunacy, 385
"——— lunatic," a misnomer, 45
——— lunatics, classification of, 389
————— employment of, 391
————— how detained, 386
————— modification of the law relating to, 387
————— suggestions for the future provision of, by W. Charles Hood, M.D., 385, 389–393
————— treatment of, 191
Criminals, mental state of, 36
——— participation of, in the profits of their work, 49
——— their age, 49
Cunning, monomaniacal, 177

Davy, J. G., on the pathology of insanity, 291
Delirium in insanity, origin of, 526
Delusion, definitions of, 412
Delusions, considered, 188
——— often founded on fact, 224
Dementia, Pinel's definition of, 32
Demonology and divination, modern, 1
Dendy, W. C., on the Pilgrimage of Thought, 80
Denham, Rev. J. F., on the connexion between morbid, physical, and religious phenomena, 639
Depletion, propriety of, considered, 227
Disease, often arrested by hope of recovery, 119
Divination, modern, 1
Drunkards, facility of reformation of, 41
Drunkenness, its effect on the mind, 40
Dynamical sympathies of the reproductive organs, 174
Dynamics, mental, in relation to the science of medicine, by M. Lordat, 252

Earl, Pliny, M.D., on American asylums, 273
Emotional sensibility, 514
Extacy, considered as a form of mental alienation, 263

Falret, M., his *Leçons Cliniques de Médecine Mentale*, 519

General paralysis, different opinions on, 55—64
Genius, the perils of, 84
German writers on logic, 65
Germany, institutions for the insane in, 443
Godfrey, Rev. N. S., on table-moving, 1—15
Guislain's work on insanity, analysis of, 263—434

Hahnemann, his doctrines, 309
Hallucinations, explanations of, considered, 529
Harrison, J. B., on the psychology of opium eating, 240
Hemiplegia, atonic, 334
——— hysterical, described, 335
Hereditary crime, 40
Hill, Frederick, on crime and its remedies, 35
——— Mr. Gardener, on the non-restraint system, 151
Holland, Asylums in, by H. Tuke, M.D., 445
Homœopathy, origin of, 307
Homicide, as an indication of insanity, 621
Hood, W. C., M.D., suggestions for the future provision of criminal lunatics, by, 385, 389, 393
Hospitals for the insane, the construction of, 297—299
Human dynamism, not illustrated by vivisection, 253
——— instincts, progressive development of, 487
Hypochondriacs, their symptoms, 321

Ideas, and their dynamic influences, 515
——— the origin of, considered, 345
Idiot, congenital, instance of, 169
Imagination, definition of, 69
Imprisonment, preferable to transportation, 46
——— silent system, considered, 46
Impulses, uncontrollable, 534
Insane, American asylums for the, 273
———autobiography of the, 356
Insane, general paralysis of the, 55
——— their hallucinations, 527—529
——— non-mechanical restraint in the treatment of the, 541
——— religious instruction of the, 103
Insanity, analysis of Guislain's work on, 263, 434
——— ancient notions of, 208
——— as affected by the sympathies, 179
——— cause of, 627
——— classification of, 222
——— criteria of, in cases of crime, 407
——— curable by medical means, 207
——— difference between the legal and medical views on, 184
——— difficulty of defining it, 27, 218, 407, 534
——— effects of sedative medicines in cases of, 233
——— general principles of medical treatment of, 226
——— incentives to, 499
——— latent, in real life, 143
——— manifestations of, how discovered, 521
——— medico-legal evidence in cases of, 184, 395, 468
——— on the increase, 388
——— pathology of, by J. G. Davey, 291
——— physical origin of, considered, 211
——— physical phenomena of, 530
——— plea of, in criminal cases, 184
——— when used, 422
——— some of the latent causes of, 159
——— statistics of, by Sir A. Morrison, M.D., 154
——— symptomatology of, 519
——— when most prevalent, 626
Insects, instinct of, 483
Instinct, considered, 484
——— independent of the intellectual principle, 255
——— of the lower animals, 483, 496
Instincts, human, 485
Intellect, how affected by the size of the cerebrum, 100
Intellectual sympathies, 183
Intuition distinguished from thought, 67
———, power of, 72
Isopathy, doctrine of, 310

Judgment, definition of, 77
——— lesion of, 525
Jurisprudence, medical, 1 84
Jury, functions of, 199

Kerslake, Roberts *v.*, case of, 572
Klepto-mania, 176
Knaggs, Samuel, on the plea of insanity in criminal cases, 184
Knowledge, intuitive, 352
Knox, Robert, M.D., F.R.S., on artistic anatomy, 504

La Salette, apparition of the Virgin at, 9
Layrock, Thomas, M.D., on the science of prevision, 1
Lettsomian Lectures, by Forbes Winslow, M.D., D.C.L., 106, 205, 395
Locke, his theory of the origin of ideas, 345
——— Psychology of, 340
——— tendency of his works, 343
Logic and Psychology, by H. L. Mansel, 64, 78
——— definition of, 67
Logical Notation, modes of, 74
Lordat, M., on mental dynamics in relation to the science of medicine, 252
Lucid intervals, 186, 197
Lunacy, recent trials in, 572
——— state of, in Paris, 289
Lunatics, subtlety of, 187
——— synopsis of dissections of, 628
Lycanthropia, 169

Magnetic magic, considered, 302
Manchester, Duke of, *v.* Bennett, case of, 598
——— Royal Lunatic Hospital, 86
Mania, as affected by blood sympathies, 167
Mansel, H. L., on Logic and Psychology, 64
Mayo, Thomas, M.D., on medical evidence in criminal cases, 184
Mechanical restraint considered, 551
———, substitutes for, 542
Medical evidence, cases in which it is needed, 406
——— Jurisprudence, 184, 468
——— Psychology, definition of, 26
——— Witnesses, duties of, 396
———, treatment of by the judges, 403
Medicine, science of, amenable to the rules of logic, 115
Meerenberg, Asylum for the Insane at, 444
Melancholia, treatment of, 235
Mental Diseases, causes and morbid anatomy of, by John Webster, M.D., F.R.S., 626
———, classification of, by M. Baillarger, 531
Mental Diseases, idiopathic and symptomatic, 539
———, principles to be followed in, 520
———, table of, 537
——— dynamics, in relation to the science of medicine, by M. Lordat, 252
——— faculties, their power, 493
——— philosophy, its study necessary to the psychological physician, 122
———, should be inductive, not speculative, 491
Mind, human, nature of, considered, 476
———, influence of, on the body, 83, 119
———, plurality of the, 82
———, unsoundness of, distinguished from insanity, 195
Monomaniacal cunning, 176
——— societies and literature, the psychology of, 301
Moral discernment, powers of, a test of insanity, 189
——— insanity, 427
Morrison, Sir A., M.D., his statistics of Insanity, 154

Necessity, Mathematical, Logical and Psychological, 77
Nervous fibres, different species of, 262
——— system, its connexion with conscious life, 511
———, its functions, 165
Noble, Daniel, on the correlation of psychology and physiology, 511
———, on the elements of psychological medicine, 24
Non-restraint system of treatment in lunacy, 151

Obstetric physician, importance of knowledge of psychology to the, 129
Opium-eating, psychology of, by J. B. Harrison, 240

Paralysis and diseases of the brain, by R. B. Todd, M.D., 326
———, hysterical, remedies recommended, 330
——— symptoms of, 328
——— of sensation and motion, different, 327
Paralytic insanity, different varieties of, 57, 59
———, progress of, 57
Paris, state of lunacy in, 289
Partial insanity, as an excuse for crime, 408
Physician, his medico-theological functions, 129

Physiology, Text-book of, by Professor Valentin, 99
——— and psychology, their correlation, 511
Poverty as an incentive to crime, 42
Prevision, the science of, by Thomas Laycock, M.D., 1
Prisoners, effect of different modes of treatment on, 52
Prisons, comparative comfort of, 49
———, their effect on young offenders, 48
Prognoses, in cases of insanity, of various kinds, 225
Prussia, institutions for the insane in, 443
Psychological adjudication, a subject for, 296
——— inquiries, 475
——— medicine, elements of, by Daniel Noble, 24
——— vocation of the physician, 106, 148
Psychology of Locke, 340
——— of monomaniacal societies and literature, the, 301
——— of opium eating, by J. B. Harrison, 240
——— and physiology, their correlation, 511
Public asylums, prejudice against, 95
Punishment, its principle, 43

Queen, the, *v.* Brough, case of, 609

Reasoning, as part of the physician's education, 114
Reproductive organs, dynamical sympathies of, 174
Responsibility, what necessary to constitute it, 421
Right and wrong, knowledge of, as a test of insanity, 471
Roberts *v.* Kerslake, case of, 572

Sedative medicine, its use in cases of insanity, 233
Skay, David, M.D., on the weight and specific gravity of the brain in insanity, 201
Sleep, want of, 499
———, its characteristics, 498
Spiritual pathology, 356
Stewart, Dugald, his theory of the connexion between mind and body, 101
St. Luke's Hospital, 300
Suicide, tendency to, considered, 216
Sympathies, the, as curative agencies in insanity, 179
———, intellectual, 183
———, responsive, 177
Syphilis, remedy for, 337

Table-moving tested, by the Rev. N. S. Godfrey, 1—13
Therapeutics, little attention paid to the study of, 205
Thought distinguished from intuition, 67
———, irritability of, 85
———, limits of, 70
———, the pilgrimage of, by W. C. Dendy, 80
———, process of, 67, 78, 80
Todd, R. B., M.D., lectures on paralysis and diseases of the brain, 326
Tuke, Dr. H., on the asylums in Holland, 445

Valentin, Professor, Text-book of Physiology, 99
Vegetable life, unconsciousness of, 494
Virchow, Rudolph, on the chemical reaction of cellulose in the brain of man, 284
Visceral sympathies described, 173, 173
"Visitors' Book" at asylums, object of, 97
Vivisection, inefficacious to illustrate the human dynamism, 253
Vivisectors, their objects, 259

Walford, Rev. Wm., autobiography of, 356
Webster, John, M.D., F.R.S., on the causes and morbid anatomy of mental diseases, 626
Will, the, its influence on the physical organism, 125
Williams, Mr. Richard, memoir of, 376
Winslow, Forbes, M.D., D.C.L., Lettsomian Lectures by, 106, 205, 395
Witnesses, medical, their duties, 396, 424
Wounds, comparative effect of, on man and animals, 255

Young, responsibility of the teachers of the, 357

Zootomists, results of their experiments on living animals, 252

